# Crystal structure of dimethyl 1-oxo-2,4-di­phenyl-1,2-dihydronaphthalene-2,3-di­carboxyl­ate

**DOI:** 10.1107/S2056989018002360

**Published:** 2018-02-13

**Authors:** Gajendran Jagadeesan, Immanuel Monica Chandramalar, Jayachandran Karunakaran, Solaiappan Gopinath, Arasambattu K. Mohanakrishnan

**Affiliations:** a Department of Physics, Jeppiaar Engineering College, Jeppiaar Nagar, OMR, Chennai 600 119, India; b Department of Organic Chemistry, University of Madras, Guindy Campus, Chennai 600 025, India; c Department of Physics, RKM Vivekananda College (Autonomous), Chennai 600 004, India

**Keywords:** crystal structure, 1,2-di­hydro­naphthalene derivative, hydrogen bonding

## Abstract

In the title compound, C_26_H_20_O_5_, a 1,2-di­hydro­naphthalene derivative, the cyclo­hexa-1,3-diene ring of the 1,2-di­hydro­naphthalene ring system adopts a half-chair conformation. The mean plane of the 1,2-di­hydro­napthalene ring system makes dihedral angles of 86.23 (6) and 64.80 (7)° with two phenyl rings. The carbonyl O atom attached to the di­hydro­naphthalene ring system deviates from the mean plane of the 1,2-di­hydro­naphthalene ring system by 0.618 (1) Å. In the crystal, the mol­ecules are linked into layers parallel to the *bc* plane *via* two kinds of C—H⋯O inter­actions, one of which forms a *C*(10) chain motif running along the *c-*axis direction and the other forms an *R* ^2^_2_(6) ring motif. Adjacent layers are further connected by C—H⋯π and offset π–π inter­actions [centroid–centroid distance = 3.6318 (9) Å].

## Abstract 2

In the title compound, a naphthalene derivative, the cyclo­hexa-1,3-diene ring of the 1,2-di­hydro­naphthalene ring system adopts a half-chair conformation. The mean plane of the 1,2-di­hydro­napthalene ring system make dihedral angles of 86.23 (6) and 64.80 (7)° with two phenyl rings. In the crystal, the mol­ecules are linked by C—H⋯O, inter­actions and further stabilized by C—H⋯π and π–π inter­actions.

## Chemical context   

Naphthalene derivatives have manifested applications in many fields, for example, as colorants, explosives, disinfectants, insecticides and the plant hormone auxin. Naphthalene is believed to play a role in the chemical defence against biological enemies (Wiltz *et al.*, 1998 ▸; Wright *et al.*, 2000 ▸). Naphthalene derivatives have been identified as a new range of potent anti-microbials that are effective against a wide range of human pathogens and have diverse and inter­esting anti­biotic properties with minimum toxicity (Rokade & Sayyed, 2009 ▸; Upadhayaya *et al.*, 2010 ▸). Compounds with non-coplanarly accumulated aromatic rings have received attention from organic chemists and materials chemists as unique structural building blocks, because they provide characteristic optical and electronic properties originating from their structural features. For example, biphenyl and binaphthyl are applied to optically active mol­ecular catalysts and polymer materials on the basis of their axial chiralities (Deria *et al.*, 2013 ▸). The structures of similar 1-oxo-1,2-di­hydro­naphtalene derivatives, namely, dimethyl 4-(4-meth­oxy­phen­yl)-2-(4-methyl­phen­yl)-1-oxo-1,2-di­hydro­naphthalene-2,3-di­carboxyl­ate, dimethyl 1-oxo-2-(pyren-4-yl)-4-(thio­phen-2-yl)-1,2-di­hydro­naphthalene-2,3-di­carboxyl­ate and ethyl 1-oxo-2-phen­yl-2,4-bis­(thio­phen-2-yl)-1,2-di­hydro­naphthalene-3-carboxyl­ate, have been reported by Gopinath *et al.* (2017 ▸).

## Structural commentary   

**Figure 1:**
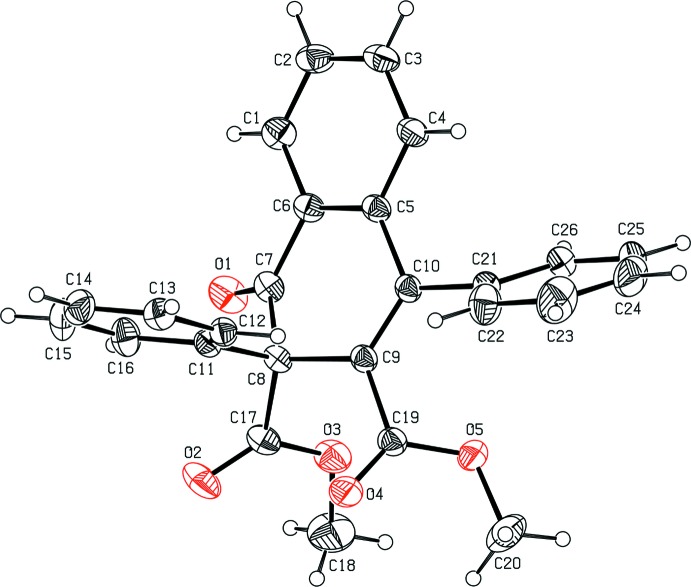
The mol­ecular structure of the title compound with the atom-numbering scheme. The displacement ellipsoids are drawn at the 30% probability level. H atoms are shown as spheres of arbitrary radii.

In the title compound (Fig. 1 ▸), the 1,2-di­hydro­naphthalene C1–C10 ring system is not strictly planar and the cyclo­hexa-1,3-diene C5–C10 ring adopts a half-chair conformation with puckering and smallest displacement parameters *q*_2_ = 0.3091 (14) Å, *q*_3_ = 0.1461 (14) Å, φ_2_ = 155.9 (3)° and θ = 64.7 (2)° and ΔC_s_ = 4.41 (19). The dihedral angle between the C1–C6 and C5–C10 rings is 10.15 (6)°. The C11–C16 phenyl ring is almost perpendicular to the 1,2-di­hydro­naphthalene C1–C10 ring system with a dihedral angle of 83.83 (7)° between them. The other phenyl ring (C21–C26) makes dihedral angles of 64.80 (7) and 29.06 (8)° with the mean plane of C1–C10 ring system and the C11–C16 phenyl ring, respectively. Atom O1 of the carbonyl group deviates from the mean plane of the 1,2-di­hydro­naphthalene ring system by 0.647 (1) Å.
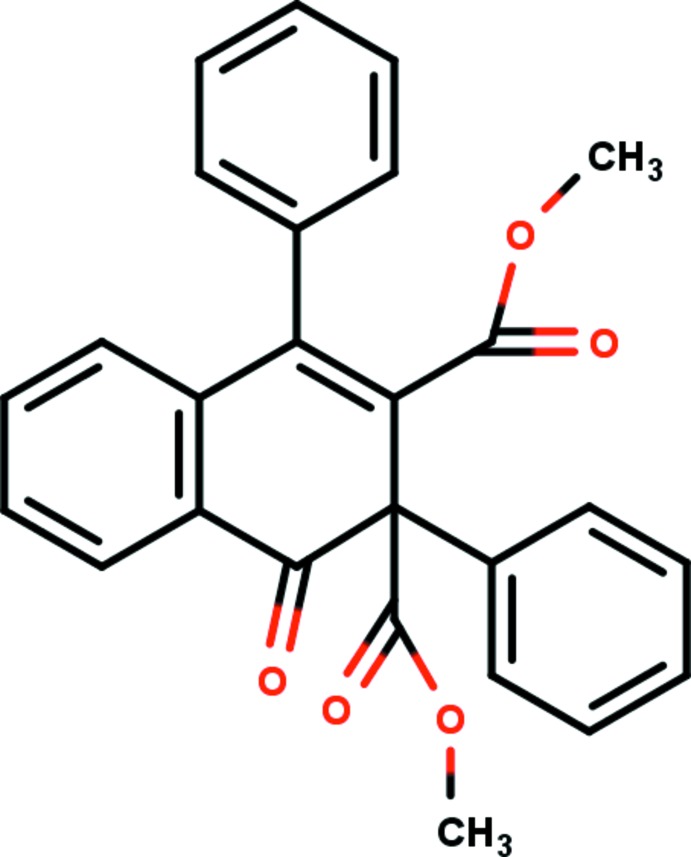


## Supra­molecular features   

**Figure 2:**
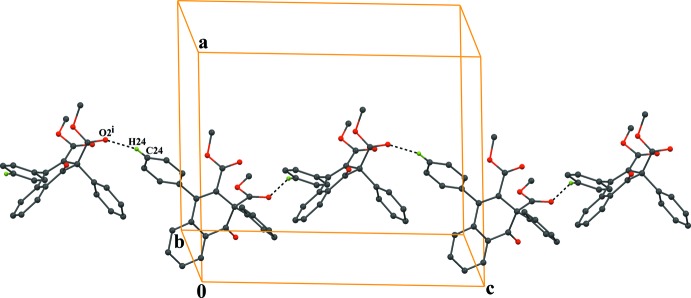
A packing diagram of the title compound, showing a *C*(10) zigzag chain along to the *c* axis formed *via* C—H⋯O hydrogen bonds (dashed lines). The H atoms not involved in the hydrogen bonding have been excluded for clarity. [Symmetry code: (i) *x*, 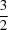 − *y*, −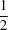 + *z*.]

**Figure 3:**
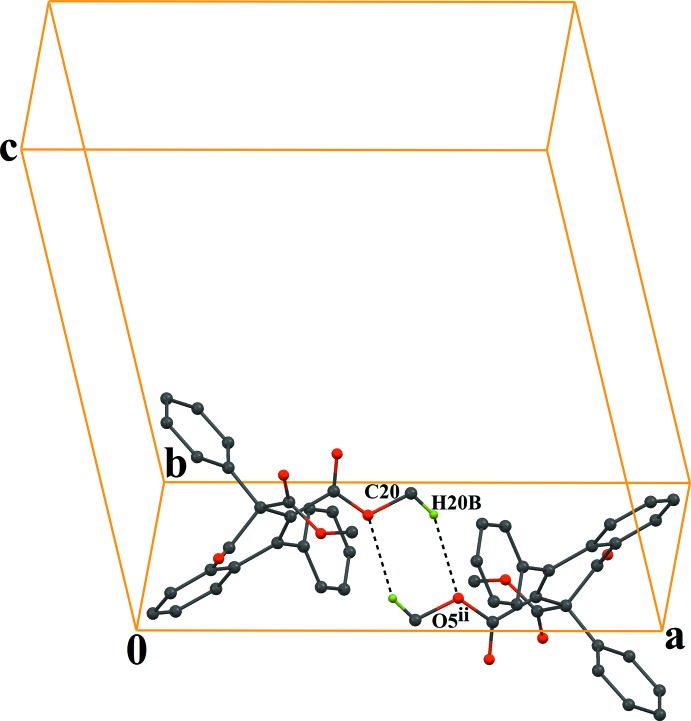
A part of the crystal packing of the title compound, showing an *R^2^_2_(6)* inversion dimer formed *via* a pair of C—H⋯O hydrogen bonds (dashed lines). The H atoms not involved in the hydrogen bonding have been excluded for clarity. [Symmetry code: (ii) 1 − *x*, 1 − *y*, −*z*.]

**Figure 4:**
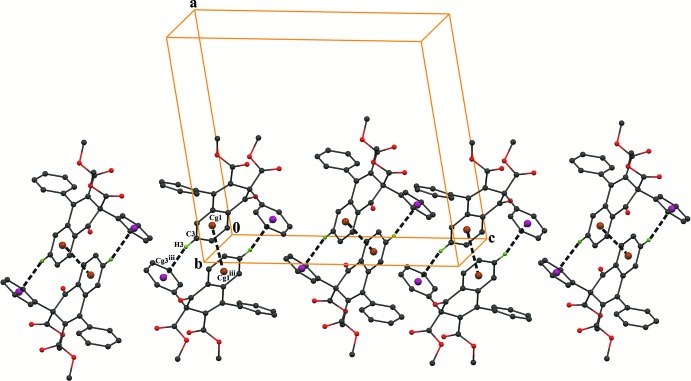
A packing diagram of the title compound, showing C—H⋯π and π–π inter­actions (dashed lines), where *Cg*1 and *Cg*3 are the centroids of the phenyl C1–C6 and C11–C16 rings, respectively. [Symmetry code: (iii) −*x*, 1 − *y*, −*z*.]

In the crystal, the mol­ecules are linked *via* C—H⋯O hydrogen bonds (C24—H24⋯O2^i^; symmetry code as in Table 1 ▸), which generates *C*(10) zigzag chains running along the *c-*axis direction (Fig. 2 ▸). In addition, the chains are linked *via* pairs of C—H⋯O inter­actions (C20—H20*B*⋯O5^ii^; Table 2 ▸) with an 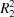
(6) ring motif (Fig. 3 ▸), forming layers parallel to the *bc* plane. Between the layers there are also C—H⋯π (C3—H3⋯*Cg*3^iii^; Table 1 ▸) and π–π stacking inter­actions (Fig. 4 ▸) [*Cg*1⋯*Cg*1^iii^ = 3.6318 (9) Å, inter­planar distance = 3.343 (1) Å and offset distance = 1.419 (1) Å; symmetry code: (iii) −*x*, 1 − *y*, −*z*; *Cg*1 and *Cg*3 are the centroids of the C1–C6 and C11–C16 rings, respectively].

## Synthesis and crystallization   

To a solution of 1,3-di­phenyl­isobenzo­furan (1 g, 3.70 mmol) in dry di­chloro­methane, dimethyl acetyl­enedi­carboxyl­ate (0.58 g, 4.07 mmol) was added and the reaction mixture was stirred at room temperature for 1 h. Removal of solvent followed by column chromatographic purification (silica gel; 15% ethyl acetate in hexa­ne) afforded isobenzo­furan­dimethyl acetyl­enedi­carboxyl­ate adduct as a colourless solid (1.10 g, 72%). To a solution of the adduct (0.50 g, 1.21 mmol) in dry di­chloro­methane, BF_3_·OEt_2_ (0.075 g, 0.52 mmol) was added and the reaction mixture was stirred at room temperature for 5 min. Removal of solvent followed by column chromatographic purification (silica gel; 15% ethyl acetate in hexa­ne) gave the title compound as a colourless solid (0.45 g, 94%). Single crystals suitable for X-ray diffraction were prepared by slow evaporation of an ethyl acetate solution of the title compound at room temperature (m.p. = 454–456 K).

## Refinement   

Crystal data, data collection and structure refinement details are summarized in Table 2 ▸. H atoms were localized in a difference-Fourier map and then were treated as riding atoms, with C—H = 0.93 and 0.96 Å for aryl and methyl groups, respectively, and with *U*_iso_(H) = 1.2*U*_eq_(aryl C) and 1.5*U*_eq_(methyl C), allowing for free rotation of the methyl groups.

## Supplementary Material

Crystal structure: contains datablock(s) I, global. DOI: 10.1107/S2056989018002360/is5487sup1.cif

Structure factors: contains datablock(s) I. DOI: 10.1107/S2056989018002360/is5487Isup2.hkl

CCDC reference: 1823056

Additional supporting information:  crystallographic information; 3D view; checkCIF report

## Figures and Tables

**Table 1:** Hydrogen-bond geometry (Å, °) *Cg*3 is the centroid of the phenyl C11–C16 ring.

*D*—H⋯*A*	*D*—H	H⋯*A*	*D*⋯*A*	*D*—H⋯*A*
C24—H24⋯O2^i^	0.93	2.59	3.449 (3)	155
C20—H20*B*⋯O5^ii^	0.96	2.59	3.430 (2)	146
C3—H3⋯*Cg*3^iii^	0.93	2.77	3.6338 (16)	154

Symmetry codes: (i) 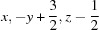; (ii) 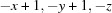; (iii) 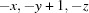.

**Table 2:** Experimental details

Crystal data	
Chemical formula	C_26_H_20_O_5_
*M*_r_	412.42
Crystal system, space group	Monoclinic, *P*2_1_/*c*
Temperature (K)	296
*a*, *b*, *c* (Å)	15.8021 (8), 7.4706 (4), 17.8599 (9)
β (°)	96.581 (2)
*V* (Å^3^)	2094.49 (19)
*Z*	4
Radiation type	Mo *K*α
μ (mm^−1^)	0.09
Crystal size (mm)	0.35 × 0.30 × 0.25

Data collection	
Diffractometer	Bruker Kappa APEXII
Absorption correction	Multi-scan (*SADABS*; Bruker, 2008 ▸)
*T*_min_, *T*_max_	0.969, 0.978
No. of measured, independent and observed [*I* > 2σ(*I*)] reflections	21819, 4614, 3375
*R*_int_	0.028
(sin θ/λ)_max_ (Å^−1^)	0.641

Refinement	
*R*[*F* ^2^ > 2σ(*F* ^2^)], *wR*(*F* ^2^), *S*	0.039, 0.108, 1.03
No. of reflections	4614
No. of parameters	283
H-atom treatment	H-atom parameters constrained
Δρ_max_, Δρ_min_ (e Å^−3^)	0.22, −0.15

Computer programs: *APEX2* and *SAINT* (Bruker, 2008 ▸), *SHELXS97* and *SHELXL97* (Sheldrick, 2008 ▸), *ORTEP-3 for Windows* (Farrugia, 2012 ▸), *Mercury* (Macrae *et al.*, 2008 ▸) and *PLATON* (Spek, 2009 ▸).

## supplementary crystallographic information

### Crystal data

**Table:**

C_26_H_20_O_5_	*F*(000) = 864
*M**_r_* = 412.42	*D*_x_ = 1.308 Mg m^−^^3^
Monoclinic, *P*2_1_/*c*	Mo *K*α radiation, λ = 0.71073 Å
Hall symbol: -P 2ybc	Cell parameters from 3375 reflections
*a* = 15.8021 (8) Å	θ = 2.3–27.1°
*b* = 7.4706 (4) Å	µ = 0.09 mm^−^^1^
*c* = 17.8599 (9) Å	*T* = 296 K
β = 96.581 (2)°	Block, colourless
*V* = 2094.49 (19) Å^3^	0.35 × 0.30 × 0.25 mm
*Z* = 4	

### Data collection

**Table:**

Bruker Kappa APEXII diffractometer	4614 independent reflections
Radiation source: fine-focus sealed tube	3375 reflections with *I* > 2σ(*I*)
Graphite monochromator	*R*_int_ = 0.028
ω & φ scans	θ_max_ = 27.1°, θ_min_ = 2.3°
Absorption correction: multi-scan (*SADABS*; Bruker, 2008)	*h* = −20→13
*T*_min_ = 0.969, *T*_max_ = 0.978	*k* = −9→8
21819 measured reflections	*l* = −22→21

### Refinement

**Table:**

Refinement on *F*^2^	Secondary atom site location: difference Fourier map
Least-squares matrix: full	Hydrogen site location: inferred from neighbouring sites
*R*[*F*^2^ > 2σ(*F*^2^)] = 0.039	H-atom parameters constrained
*wR*(*F*^2^) = 0.108	*w* = 1/[σ^2^(*F*_o_^2^) + (0.0478*P*)^2^ + 0.4036*P*] where *P* = (*F*_o_^2^ + 2*F*_c_^2^)/3
*S* = 1.03	(Δ/σ)_max_ = 0.017
4614 reflections	Δρ_max_ = 0.22 e Å^−^^3^
283 parameters	Δρ_min_ = −0.14 e Å^−^^3^
0 restraints	Extinction correction: SHELXL, Fc^*^=kFc[1+0.001xFc^2^λ^3^/sin(2θ)]^-1/4^
Primary atom site location: structure-invariant direct methods	Extinction coefficient: 0.0033 (8)

### Special details

**Table:**

Geometry. All esds (except the esd in the dihedral angle between two l.s. planes) are estimated using the full covariance matrix. The cell esds are taken into account individually in the estimation of esds in distances, angles and torsion angles; correlations between esds in cell parameters are only used when they are defined by crystal symmetry. An approximate (isotropic) treatment of cell esds is used for estimating esds involving l.s. planes.
Refinement. Refinement of F^2^ against ALL reflections. The weighted R-factor wR and goodness of fit S are based on F^2^, conventional R-factors R are based on F, with F set to zero for negative F^2^. The threshold expression of F^2^ > 2sigma(F^2^) is used only for calculating R-factors(gt) etc. and is not relevant to the choice of reflections for refinement. R-factors based on F^2^ are statistically about twice as large as those based on F, and R- factors based on ALL data will be even larger.

### Fractional atomic coordinates and isotropic or equivalent isotropic displacement parameters (Å^2^)

**Table:**

	*x*	*y*	*z*	*U*_iso_*/*U*_eq_	
C1	0.05662 (10)	0.2111 (2)	0.01264 (8)	0.0463 (4)	
H1	0.0388	0.1135	0.0391	0.056*	
C2	0.00596 (10)	0.2750 (2)	−0.04963 (8)	0.0512 (4)	
H2	−0.0464	0.2217	−0.0648	0.061*	
C3	0.03304 (10)	0.4172 (2)	−0.08905 (8)	0.0488 (4)	
H3	−0.0011	0.4600	−0.1311	0.059*	
C4	0.11058 (9)	0.4978 (2)	−0.06695 (7)	0.0433 (3)	
H4	0.1284	0.5932	−0.0947	0.052*	
C5	0.16244 (8)	0.43761 (18)	−0.00354 (7)	0.0354 (3)	
C6	0.13411 (8)	0.29249 (18)	0.03586 (7)	0.0368 (3)	
C7	0.18723 (9)	0.22355 (19)	0.10317 (7)	0.0384 (3)	
C8	0.24887 (8)	0.35947 (17)	0.14498 (6)	0.0338 (3)	
C9	0.28722 (8)	0.48058 (18)	0.08927 (7)	0.0336 (3)	
C10	0.24565 (8)	0.52183 (18)	0.02146 (7)	0.0338 (3)	
C11	0.19360 (8)	0.46192 (18)	0.19635 (6)	0.0344 (3)	
C12	0.18236 (8)	0.64551 (18)	0.19151 (7)	0.0368 (3)	
H12	0.2110	0.7110	0.1580	0.044*	
C13	0.12938 (9)	0.7325 (2)	0.23568 (8)	0.0447 (3)	
H13	0.1228	0.8560	0.2319	0.054*	
C14	0.08630 (10)	0.6384 (2)	0.28520 (8)	0.0530 (4)	
H14	0.0499	0.6971	0.3145	0.064*	
C15	0.09745 (11)	0.4569 (3)	0.29111 (9)	0.0595 (5)	
H15	0.0691	0.3926	0.3252	0.071*	
C16	0.15022 (10)	0.3687 (2)	0.24720 (8)	0.0505 (4)	
H16	0.1568	0.2453	0.2517	0.061*	
C17	0.31896 (9)	0.24874 (19)	0.19065 (8)	0.0430 (3)	
C18	0.44438 (13)	0.0867 (3)	0.17834 (12)	0.0857 (7)	
H18A	0.4743	0.1552	0.2185	0.129*	
H18B	0.4820	0.0603	0.1412	0.129*	
H18C	0.4246	−0.0231	0.1982	0.129*	
C19	0.37168 (9)	0.55771 (19)	0.11799 (7)	0.0387 (3)	
C20	0.50781 (11)	0.6419 (3)	0.08805 (11)	0.0783 (6)	
H20A	0.5019	0.7671	0.0983	0.117*	
H20B	0.5429	0.6268	0.0480	0.117*	
H20C	0.5338	0.5825	0.1325	0.117*	
C21	0.27693 (8)	0.6601 (2)	−0.02840 (7)	0.0398 (3)	
C22	0.28407 (11)	0.8357 (2)	−0.00451 (10)	0.0593 (4)	
H22	0.2708	0.8659	0.0433	0.071*	
C23	0.31073 (13)	0.9673 (3)	−0.05105 (14)	0.0824 (6)	
H23	0.3156	1.0851	−0.0344	0.099*	
C24	0.33002 (12)	0.9242 (4)	−0.12164 (13)	0.0846 (7)	
H24	0.3474	1.0127	−0.1531	0.102*	
C25	0.32355 (11)	0.7507 (4)	−0.14553 (9)	0.0723 (6)	
H25	0.3375	0.7214	−0.1932	0.087*	
C26	0.29671 (9)	0.6184 (3)	−0.10003 (8)	0.0531 (4)	
H26	0.2918	0.5010	−0.1173	0.064*	
O1	0.18036 (7)	0.07330 (14)	0.12689 (6)	0.0561 (3)	
O2	0.32467 (8)	0.21704 (16)	0.25637 (6)	0.0629 (3)	
O3	0.37245 (7)	0.18860 (15)	0.14387 (6)	0.0548 (3)	
O4	0.38995 (7)	0.60087 (17)	0.18244 (5)	0.0600 (3)	
O5	0.42502 (6)	0.56566 (15)	0.06595 (5)	0.0508 (3)	

### Atomic displacement parameters (Å^2^)

**Table:**

	*U*^11^	*U*^22^	*U*^33^	*U*^12^	*U*^13^	*U*^23^
C1	0.0517 (8)	0.0388 (8)	0.0475 (8)	−0.0064 (7)	0.0016 (6)	−0.0088 (6)
C2	0.0441 (8)	0.0553 (10)	0.0514 (8)	−0.0034 (7)	−0.0068 (7)	−0.0165 (7)
C3	0.0473 (8)	0.0566 (10)	0.0394 (7)	0.0071 (7)	−0.0084 (6)	−0.0076 (7)
C4	0.0447 (8)	0.0500 (9)	0.0340 (7)	0.0043 (7)	−0.0011 (6)	0.0003 (6)
C5	0.0384 (7)	0.0373 (8)	0.0300 (6)	0.0046 (6)	0.0016 (5)	−0.0049 (5)
C6	0.0422 (7)	0.0334 (7)	0.0338 (6)	0.0010 (6)	0.0006 (5)	−0.0076 (5)
C7	0.0466 (8)	0.0326 (8)	0.0359 (7)	0.0017 (6)	0.0044 (6)	−0.0022 (6)
C8	0.0398 (7)	0.0326 (7)	0.0281 (6)	0.0019 (5)	−0.0006 (5)	0.0004 (5)
C9	0.0361 (6)	0.0361 (7)	0.0288 (6)	0.0026 (5)	0.0041 (5)	−0.0029 (5)
C10	0.0362 (6)	0.0370 (7)	0.0285 (6)	0.0045 (6)	0.0052 (5)	−0.0026 (5)
C11	0.0385 (7)	0.0366 (8)	0.0275 (6)	−0.0022 (6)	0.0013 (5)	−0.0001 (5)
C12	0.0380 (7)	0.0380 (8)	0.0342 (6)	−0.0032 (6)	0.0041 (5)	0.0007 (5)
C13	0.0464 (8)	0.0421 (9)	0.0454 (8)	0.0010 (6)	0.0047 (6)	−0.0078 (6)
C14	0.0490 (9)	0.0658 (12)	0.0463 (8)	−0.0050 (8)	0.0146 (7)	−0.0155 (8)
C15	0.0701 (11)	0.0657 (12)	0.0475 (9)	−0.0119 (9)	0.0272 (8)	0.0014 (8)
C16	0.0668 (10)	0.0416 (9)	0.0452 (8)	−0.0066 (7)	0.0155 (7)	0.0035 (7)
C17	0.0506 (8)	0.0381 (8)	0.0383 (7)	0.0050 (6)	−0.0036 (6)	0.0008 (6)
C18	0.0694 (13)	0.0904 (16)	0.0951 (15)	0.0431 (11)	0.0000 (11)	0.0129 (12)
C19	0.0384 (7)	0.0431 (8)	0.0344 (7)	0.0035 (6)	0.0030 (5)	−0.0026 (6)
C20	0.0424 (9)	0.1093 (17)	0.0855 (13)	−0.0188 (10)	0.0176 (9)	−0.0311 (12)
C21	0.0363 (7)	0.0489 (9)	0.0343 (7)	0.0033 (6)	0.0039 (5)	0.0079 (6)
C22	0.0679 (11)	0.0505 (11)	0.0622 (10)	0.0009 (8)	0.0191 (8)	0.0096 (8)
C23	0.0831 (14)	0.0603 (13)	0.1064 (17)	−0.0032 (10)	0.0224 (12)	0.0306 (12)
C24	0.0596 (12)	0.1074 (19)	0.0883 (15)	−0.0008 (12)	0.0150 (10)	0.0621 (14)
C25	0.0470 (9)	0.128 (2)	0.0429 (9)	0.0034 (11)	0.0090 (7)	0.0312 (11)
C26	0.0438 (8)	0.0809 (12)	0.0347 (7)	0.0026 (8)	0.0045 (6)	0.0059 (7)
O1	0.0767 (8)	0.0338 (6)	0.0554 (6)	−0.0059 (5)	−0.0029 (5)	0.0052 (5)
O2	0.0806 (8)	0.0670 (8)	0.0383 (6)	0.0193 (6)	−0.0058 (5)	0.0116 (5)
O3	0.0531 (6)	0.0581 (7)	0.0521 (6)	0.0213 (5)	0.0011 (5)	−0.0005 (5)
O4	0.0472 (6)	0.0932 (9)	0.0387 (5)	−0.0121 (6)	0.0013 (4)	−0.0186 (6)
O5	0.0362 (5)	0.0741 (8)	0.0430 (5)	−0.0041 (5)	0.0081 (4)	−0.0076 (5)

### Geometric parameters (Å, º)

**Table:**

C1—C2	1.379 (2)	C14—H14	0.9300
C1—C6	1.3873 (19)	C15—C16	1.376 (2)
C1—H1	0.9300	C15—H15	0.9300
C2—C3	1.370 (2)	C16—H16	0.9300
C2—H2	0.9300	C17—O2	1.1905 (16)
C3—C4	1.381 (2)	C17—O3	1.3328 (18)
C3—H3	0.9300	C18—O3	1.4456 (19)
C4—C5	1.3942 (17)	C18—H18A	0.9600
C4—H4	0.9300	C18—H18B	0.9600
C5—C6	1.3940 (19)	C18—H18C	0.9600
C5—C10	1.4796 (18)	C19—O4	1.1985 (15)
C6—C7	1.4777 (18)	C19—O5	1.3256 (17)
C7—O1	1.2090 (17)	C20—O5	1.4399 (19)
C7—C8	1.5397 (18)	C20—H20A	0.9600
C8—C9	1.5212 (18)	C20—H20B	0.9600
C8—C17	1.5392 (17)	C20—H20C	0.9600
C8—C11	1.5408 (18)	C21—C22	1.380 (2)
C9—C10	1.3457 (16)	C21—C26	1.3867 (19)
C9—C19	1.4897 (18)	C22—C23	1.384 (2)
C10—C21	1.4852 (18)	C22—H22	0.9300
C11—C12	1.3844 (19)	C23—C24	1.369 (3)
C11—C16	1.3866 (19)	C23—H23	0.9300
C12—C13	1.3774 (19)	C24—C25	1.365 (3)
C12—H12	0.9300	C24—H24	0.9300
C13—C14	1.371 (2)	C25—C26	1.377 (3)
C13—H13	0.9300	C25—H25	0.9300
C14—C15	1.369 (2)	C26—H26	0.9300
			
C2—C1—C6	120.04 (15)	C14—C15—C16	120.74 (15)
C2—C1—H1	120.0	C14—C15—H15	119.6
C6—C1—H1	120.0	C16—C15—H15	119.6
C3—C2—C1	119.74 (14)	C15—C16—C11	120.65 (15)
C3—C2—H2	120.1	C15—C16—H16	119.7
C1—C2—H2	120.1	C11—C16—H16	119.7
C2—C3—C4	120.68 (13)	O2—C17—O3	124.69 (13)
C2—C3—H3	119.7	O2—C17—C8	126.72 (14)
C4—C3—H3	119.7	O3—C17—C8	108.57 (11)
C3—C4—C5	120.74 (14)	O3—C18—H18A	109.5
C3—C4—H4	119.6	O3—C18—H18B	109.5
C5—C4—H4	119.6	H18A—C18—H18B	109.5
C6—C5—C4	117.90 (12)	O3—C18—H18C	109.5
C6—C5—C10	120.27 (11)	H18A—C18—H18C	109.5
C4—C5—C10	121.82 (12)	H18B—C18—H18C	109.5
C1—C6—C5	120.89 (12)	O4—C19—O5	123.97 (13)
C1—C6—C7	119.35 (13)	O4—C19—C9	122.85 (13)
C5—C6—C7	119.76 (12)	O5—C19—C9	113.11 (11)
O1—C7—C6	122.90 (12)	O5—C20—H20A	109.5
O1—C7—C8	121.30 (11)	O5—C20—H20B	109.5
C6—C7—C8	115.69 (11)	H20A—C20—H20B	109.5
C9—C8—C17	110.48 (11)	O5—C20—H20C	109.5
C9—C8—C7	110.63 (10)	H20A—C20—H20C	109.5
C17—C8—C7	106.22 (10)	H20B—C20—H20C	109.5
C9—C8—C11	112.92 (10)	C22—C21—C26	118.68 (14)
C17—C8—C11	111.96 (10)	C22—C21—C10	119.79 (12)
C7—C8—C11	104.24 (10)	C26—C21—C10	121.49 (14)
C10—C9—C19	123.16 (12)	C21—C22—C23	120.60 (17)
C10—C9—C8	122.34 (11)	C21—C22—H22	119.7
C19—C9—C8	114.40 (10)	C23—C22—H22	119.7
C9—C10—C5	119.96 (12)	C24—C23—C22	120.1 (2)
C9—C10—C21	122.49 (12)	C24—C23—H23	119.9
C5—C10—C21	117.40 (10)	C22—C23—H23	119.9
C12—C11—C16	117.97 (13)	C25—C24—C23	119.64 (18)
C12—C11—C8	122.16 (11)	C25—C24—H24	120.2
C16—C11—C8	119.81 (12)	C23—C24—H24	120.2
C13—C12—C11	120.92 (13)	C24—C25—C26	120.94 (18)
C13—C12—H12	119.5	C24—C25—H25	119.5
C11—C12—H12	119.5	C26—C25—H25	119.5
C14—C13—C12	120.45 (14)	C25—C26—C21	120.02 (18)
C14—C13—H13	119.8	C25—C26—H26	120.0
C12—C13—H13	119.8	C21—C26—H26	120.0
C15—C14—C13	119.25 (14)	C17—O3—C18	115.77 (13)
C15—C14—H14	120.4	C19—O5—C20	117.16 (12)
C13—C14—H14	120.4		
			
C6—C1—C2—C3	1.0 (2)	C9—C8—C11—C16	176.27 (12)
C1—C2—C3—C4	−0.1 (2)	C17—C8—C11—C16	−58.27 (16)
C2—C3—C4—C5	−0.8 (2)	C7—C8—C11—C16	56.13 (14)
C3—C4—C5—C6	1.0 (2)	C16—C11—C12—C13	−0.28 (18)
C3—C4—C5—C10	−179.82 (13)	C8—C11—C12—C13	176.90 (12)
C2—C1—C6—C5	−0.8 (2)	C11—C12—C13—C14	−0.3 (2)
C2—C1—C6—C7	179.38 (13)	C12—C13—C14—C15	1.0 (2)
C4—C5—C6—C1	−0.2 (2)	C13—C14—C15—C16	−1.0 (2)
C10—C5—C6—C1	−179.37 (12)	C14—C15—C16—C11	0.4 (2)
C4—C5—C6—C7	179.65 (12)	C12—C11—C16—C15	0.2 (2)
C10—C5—C6—C7	0.44 (18)	C8—C11—C16—C15	−177.00 (13)
C1—C6—C7—O1	21.7 (2)	C9—C8—C17—O2	138.57 (16)
C5—C6—C7—O1	−158.09 (14)	C7—C8—C17—O2	−101.40 (17)
C1—C6—C7—C8	−154.54 (12)	C11—C8—C17—O2	11.8 (2)
C5—C6—C7—C8	25.65 (18)	C9—C8—C17—O3	−42.97 (14)
O1—C7—C8—C9	145.22 (13)	C7—C8—C17—O3	77.06 (14)
C6—C7—C8—C9	−38.46 (15)	C11—C8—C17—O3	−169.76 (11)
O1—C7—C8—C17	25.29 (17)	C10—C9—C19—O4	140.82 (15)
C6—C7—C8—C17	−158.39 (11)	C8—C9—C19—O4	−35.58 (19)
O1—C7—C8—C11	−93.12 (15)	C10—C9—C19—O5	−42.04 (18)
C6—C7—C8—C11	83.21 (13)	C8—C9—C19—O5	141.56 (12)
C17—C8—C9—C10	146.53 (12)	C9—C10—C21—C22	−62.17 (19)
C7—C8—C9—C10	29.18 (17)	C5—C10—C21—C22	113.49 (15)
C11—C8—C9—C10	−87.22 (15)	C9—C10—C21—C26	120.02 (15)
C17—C8—C9—C19	−37.04 (15)	C5—C10—C21—C26	−64.33 (17)
C7—C8—C9—C19	−154.38 (11)	C26—C21—C22—C23	−0.2 (2)
C11—C8—C9—C19	89.22 (13)	C10—C21—C22—C23	−178.11 (15)
C19—C9—C10—C5	179.15 (12)	C21—C22—C23—C24	0.3 (3)
C8—C9—C10—C5	−4.73 (19)	C22—C23—C24—C25	−0.6 (3)
C19—C9—C10—C21	−5.3 (2)	C23—C24—C25—C26	0.9 (3)
C8—C9—C10—C21	170.82 (12)	C24—C25—C26—C21	−0.9 (2)
C6—C5—C10—C9	−11.87 (19)	C22—C21—C26—C25	0.6 (2)
C4—C5—C10—C9	168.95 (13)	C10—C21—C26—C25	178.39 (13)
C6—C5—C10—C21	172.36 (12)	O2—C17—O3—C18	−3.7 (2)
C4—C5—C10—C21	−6.82 (18)	C8—C17—O3—C18	177.83 (14)
C9—C8—C11—C12	−0.86 (16)	O4—C19—O5—C20	−4.2 (2)
C17—C8—C11—C12	124.60 (13)	C9—C19—O5—C20	178.73 (14)
C7—C8—C11—C12	−121.00 (12)		

### Hydrogen-bond geometry (Å, º)

*Cg*3 is the centroid of the phenyl C11–C16 ring.

**Table:**

*D*—H···*A*	*D*—H	H···*A*	*D*···*A*	*D*—H···*A*
C24—H24···O2^i^	0.93	2.59	3.449 (3)	155
C20—H20*B*···O5^ii^	0.96	2.59	3.430 (2)	146
C3—H3···*Cg*3^iii^	0.93	2.77	3.6338 (16)	154

Symmetry codes: (i) *x*, −*y*+3/2, *z*−1/2; (ii) −*x*+1, −*y*+1, −*z*; (iii) −*x*, −*y*+1, −*z*.
